# What Drives National Differences in Intensive Grandparental Childcare in Europe?

**DOI:** 10.1093/geronb/gbv007

**Published:** 2015-03-16

**Authors:** Giorgio Di Gessa, Karen Glaser, Debora Price, Eloi Ribe, Anthea Tinker

**Affiliations:** Department of Social Science, Health and Medicine, Institute of Gerontology, School of Social Science and Public Policy, King’s College London, The Strand, London WC2R 2LS, UK.

**Keywords:** Europe, Grandparents, Childcare, Female labor force participation, Intergenerational relationships, SHARE.

## Abstract

**Objectives.:**

Grandparents play an important role in looking after grandchildren, although intensive grandparental childcare varies considerably across Europe. Few studies have explicitly investigated the extent to which such cross-national variations are associated with national level differences in individual demographic and socio-economic distributions along with contextual-structural and cultural factors (e.g., variations in female labor force participation, childcare provision, and cultural attitudes).

**Methods.:**

We used multilevel models to examine associations between intensive grandparental childcare and contextual-structural and cultural factors, after controlling for grandparent, parent, and child characteristics using nationally representative data from the Survey of Health, Ageing and Retirement in Europe.

**Results.:**

Even controlling for cross-national differences in demographic and socio-economic distributions, contextual-structural factors play an important role in explaining grandparental childcare variations in Europe. In particular, higher levels of intensive grandparental childcare are found in countries with low labor force participation among younger and older women, and low formal childcare provision, where mothers in paid work largely rely on grandparental support on an almost daily basis.

**Discussion.:**

Encouraging older women to remain in paid work is likely to have an impact on grandchild care which in turn may affect mothers’ employment, particularly in Southern European countries where there is little formal childcare.

Across Europe increased life expectancy means that it is now quite common for children to grow up while their grandparents and even great grandparents are still living (Murphy, 2011; Post, Van Poppel, Van Imhoff, & Kruse, 1997). Aging populations, and other socio-demographic changes such as more mothers in the labor market and higher levels of divorce and separation, suggest that grandparents are likely to play an increasingly significant role in family life (Aassve, Arpino, & Goisis, 2012; Herlofson & Hagestad, 2012; King, 2003; Wheelock & Jones, 2002). Particularly, the role that grandparents play in providing childcare is attracting increasing academic and policy attention.

A substantial body of work, especially in the US, has investigated individual and family demographic and socio-economic characteristics of both the provider and recipient of grandparental childcare (Arpino, Pronzato, & Tavares, 2010; Baydar & Brooks-Gunn, 1998; Fuller-Thomson & Minkler, 2001; Glaser et al., 2013; Hagestad, 2006; Hank & Buber, 2009; Herlofson & Hagestad, 2012; King, 2003; Koslowski, 2009; Minkler & Fuller-Thomson, 2005; Vandell, McCartney, Owen, Booth, & Clarke-Stewart, 2003; Wheelock & Jones, 2002; Zamarro, 2011). Recent European comparative research shows significant national differences in the level of grandparent childcare, after controlling for characteristics of grandparents, parents, and children. However, in this literature whether contextual-structural and cultural factors—such as the labor market, formal childcare provision, and attitudes toward formal childcare—may help to explain cross-national variations in grandchild care has received less attention (Albertini, Kohli, & Vogel, 2007; Igel & Szydlik, 2011; Jappens & van Bavel, 2012).

Europe represents a unique setting for examining intergenerational childcare as it is recognised that factors such as provision of services and generosity of child benefits; pension schemes; and labor, retirement, and early-retirement policies; as well as cultural norms and values vary considerably (Arts & Gelissen, 2002). In feminist work on family policy, comparative research has illustrated the important role played by macroinstitutional and cultural contexts in shaping mothers’ care and employment relations, especially policy environments, cultural norms, and historic trajectories (Daly, 2000; Keck & Saraceno, 2013; Nieuwenhuis, Need, & Van Der Kolk, 2012; Orloff, 2002). This large body of work has heavily focussed on mothers yet we should expect similar factors to influence grandparental care which is largely undertaken by maternal grandparents, and taking place in similarly varying economic, employment, and cultural contexts. So far, despite the policy implications, few scholars have attempted to understand how these important factors affect this system of wider family care, where, essentially, two generations of mothers are involved. Thus, very little is known about whether observed variation in patterns of grandparental childcare is primarily a result of differences across countries in the distribution of key individual demographic and socio-economic characteristics (such as the ages of grandparents or numbers of grandchildren) which are also known to vary widely from country to country, or whether policy driven contexts such as the operation of labor markets and childcare facilities are the main drivers. Our research aims to examine whether national differences in grandparental childcare observed in selected European countries are largely demographically driven or whether they are accounted for by country-specific contexts reflecting women’s participation in the labor market, levels of formal childcare provision and cultural attitudes toward formal childcare. Thus, our study provides a valuable contribution to the discussion of the effects that policy-driven structures and values may have on one particular type of intergenerational transfer: provision of grandchild care.

## Background

Grandparents play an active role in the lives of their grandchildren. In the United States, 24% of children under five have been cared for by grandparents in the previous month (Laughlin, 2013), and a study of 11 European countries showed that 58% of grandmothers and 49% of grandfathers looked after at least one of their grandchildren aged under 16 in the preceding year in the absence of parents (Hank & Buber, 2009). Nevertheless, there are striking national differences in the frequency of grandparental childcare. The probability of providing any grandparental childcare is generally higher in Denmark, Sweden, the Netherlands, and France (around 60%) than in the Southern European countries (less than 50%). Yet when grandparents in Southern European countries do provide childcare, they do so more regularly (i.e., almost weekly or more often) (Albertini, Kohli, & Vogel, 2007; Hank & Buber, 2009; Igel & Szydlik, 2011). In Britain, 63% of grandparents provided some childcare to grandchildren under 16, 17% providing at least 10hr a week (Wellard, 2011).

The literature investigating individual characteristics associated with grandparental childcare is extensive. Both grandparents contribute to informal childcare, although grandmothers are more likely to provide care, maternal grandmothers in particular (Fuller-Thomson & Minkler, 2001; Minkler & Fuller-Thomson, 2005; Wheelock & Jones, 2002). Younger and healthier grandparents are more likely to look after their grandchildren (Baydar & Brooks-Gunn, 1998; Glaser et al., 2013; King, 2003), particularly if they are not working (Fuller-Thomson & Minkler, 2001; Hank & Buber, 2009; Igel & Szydlik, 2011; Zamarro, 2011), and grandparents (particularly grandfathers) are less likely to look after grandchildren if they live alone (Hank & Buber, 2009). Evidence on the association between financial resources and grandchild care is mixed and depends on the intensity of care (Vandell et al., 2003). Grandparents with “primary care” responsibilities for grandchildren are more likely to be disadvantaged, have lower educational attainment and poor (Fuller-Thomson & Minkler, 2001; Minkler & Fuller-Thomson, 2005); grandparents providing occasional or regular childcare are generally financially better-off (Albertini, Kohli, & Vogel, 2007; Baydar & Brooks-Gunn, 1998; Hank & Buber, 2009; Igel & Szydlik, 2011).

Parents’ characteristics are also associated with grandparental childcare. Younger parents (especially mothers), those in paid work, and those separated or divorced are all more likely to use grandparent childcare (Herlofson & Hagestad, 2012; Koslowski, 2009; Vandell et al., 2003; Wheelock & Jones, 2002; Zamarro, 2011); and Arpino et al. (2010) have shown that in Italy and France mothers are more likely to engage in paid work when grandparents provide childcare. Family size and the ages of grandchildren are also important: Parents with siblings have less help with care from grandparents, possibly because grandparents with more children are more likely to have more grandchildren, limiting the amount of support to each (Aassve, Meroni, & Pronzato, 2012). Also, some grandparental childcare is more likely if grandchildren are aged four to six, with regular childcare more likely for children under three (Igel & Szydlik, 2011).

Although the studies mentioned above examined the relationship between individual-level characteristics and grandparental childcare, they did not explicitly consider whether national distributions in key demographic and socio-economic characteristics explain cross-national variation in grandparental care. This is important because grandparents in Southern Mediterranean countries may be looking after grandchildren more regularly because they have fewer grandchildren compared with grandparents in Scandinavian countries (Glaser et al., 2013).

### Contextual-Structural Factors

In addition to variations in the distribution of individual demographic and socio-economic characteristics, European countries also differ in terms of policies, contextual-structural, and cultural factors including welfare state provision, structural labor market constraints, formal childcare provision, and family norms. To understand grandparental childcare provision, we need to study not only parents’ and grandparents’ characteristics but also country-level contextual factors that may help to explain these variations. Since family policy research has shown the critical association between mother’s employment and parental childcare arrangements (Daly, 2000; Keck & Saraceno, 2013; Nieuwenhuis, Need, & Van Der Kolk, 2012; Orloff, 2002), understanding the institutional and cultural environment for grandparental employment (and grandmothers’ employment in particular) is likely to provide a key part of the explanation for variation in grandparental childcare. Grandmaternal employment may interact with mother’s employment to explain patterns and cultures of care, and this may be especially important in countries which have experienced large generational shifts in patterns of women’s employment in recent decades. Understanding the institutional employment structures and cultures for women of different ages would seem therefore to be an important part of this discussion.

Many studies have shown that the availability of childcare is an important factor in determining mother’s employment and maternal childcare (Keck & Saraceno, 2013). Studies on grandparental childcare have suggested that parents are less likely to rely on grandparents in those countries with greater provision of formal childcare (Attias-Donfut, Ogg, & Wolff, 2005; Hank & Buber, 2009; Koslowski, 2009). However only one study has formally tested this: Igel and Szydlik (2011) show that in those European countries with low national expenditure on family benefits and formal childcare, more grandparents provide grandchild care at least weekly.

We know far less about the associations between women’s participation in the labor market, cultural attitudes toward mothers’ care, and grandparental childcare. Although several studies suggest that female or maternal employment regimes in Europe may help to explain observed variations in the prevalence of grandparental childcare, again none of these studies formally tested these country-level indicators (Attias-Donfut, Ogg, & Wolff, 2005; Hank & Buber, 2009; Koslowski, 2009). Furthermore, to date, only one study has attempted to investigate the role of cultural norms. Jappens and van Bavel (2012) show that mothers with children under age 12 are more likely to use grandparents as the main source of childcare in European regions with more conservative attitudes toward gendered family roles.

Thus, few studies have attempted to directly measure how individual, contextual-structural, and cultural level factors in combination may influence the role grandparents play in family life (Albertini, Kohli, & Vogel, 2007; Igel & Szydlik, 2011; Jappens & van Bavel, 2012). Even though the availability of and attitudes toward formal childcare and labor market structures for the recipients and the providers of childcare are likely to be related (Daly, 2000; Keck & Saraceno, 2013; Nieuwenhuis, Need, & Van Der Kolk, 2012; Orloff, 2002), no study has looked at these factors simultaneously.

Glaser and colleagues (2013) thus hypothesise that the degree to which grandmothers look after grandchildren should depend not only on the provision of formal childcare and on cultural norms about care and family obligations, but also on the extent to which mothers and grandmothers participate in the labor market, which varies widely from country to country. Little prior academic thought has been given to what the expectation might be for grandparental care (and grandmaternal care in particular) if both institutional (employment and childcare) and cultural factors suggest that mothers are expected to care intensively for their children, especially young children. Glaser and colleagues (2013) suggest that lower levels of grandparental childcare might be expected in those countries where rates of female employment are high, because formal childcare structures are better; however, in countries where a high percentage of mothers do not work and where family care is preferred and formal childcare is limited, mothers who do not conform to the expected pattern, especially those who work full time, might have a very high need for grandparental childcare, in turn influenced by the structural availability of grandmothers to provide the care needed.

Thus, our study aims to investigate the extent to which variation in patterns of grandparental care in Europe can be explained by national demographic and socio-economic differences between individuals, and by structural and cultural factors. These are operationalised following Glaser and colleagues (2013) by the labor market participation rates of different generations of women (the percentage of women 50–64 in paid work, the percentage of mothers aged 25–49 not in paid work), formal childcare use (the percentage of children aged 0–2 enrolled in formal childcare), and national attitudes toward maternal childcare for young children. It thus uniquely approaches grandparental childcare using both micro and macrolevel indicators and following Hagestad (2006) we also consider an intergenerational perspective including characteristics of grandparents, parents, and grandchildren simultaneously. Our focus here is on intensive grandparental childcare, as this type of childcare is most likely to be influenced by macroindicators and the employment rates of mid-life women in contrast to more sporadic care (Vandell et al., 2003), as well as having potentially the most important policy implications.

## Method

### Study Population

We used data from SHARE, a biennial longitudinal survey designed to enable comparative analyses across 11 European countries, namely Sweden, Denmark, Germany, the Netherlands, France, Belgium, Austria, Switzerland, Italy, Spain, and Greece. SHARE aimed to be representative of the relevant national populations aged 50 and over. It has an (unweighted) average household response rate of 62%, ranging from 39% in Belgium and Switzerland to 81% in France. Among baseline respondents almost a third (32%) have dropped out of the study (with attrition as high as 48% in Germany); research has shown that such attrition is unlikely to be random (Fitzgerald, Gottschalk, & Moffit, 1998). We therefore decided to base our study on the first survey wave which took place in 2004/2005, as data quality checks have shown that baseline data are broadly representative of national populations (Börsch-Supan & Jürges, 2005). Further details on the sampling frames and methodology, weighting strategies, and questionnaires are available elsewhere (Börsch-Supan & Jürges, 2005).

SHARE provides information on the socio-economic, health, and demographic characteristics of individuals aged 50 and over. It also includes comprehensive information about the frequency and intensity of grandparental childcare, characteristics of the respondents’ adult children and ages of grandchildren. Wave 1 is based on 27,520 total respondents. We restricted our sample to respondents aged 50 and over with at least one grandchild (*N* = 16,510, 60% of total initial respondents). Respondents provide detailed information (such as gender, age, employment, and marital status) for up to four living children. If respondents had more than four children, only information on those who lived closer and/or those who were older were collected. Our analysis was thus restricted to grandparents with adult children living in a separate, private household, and whose own youngest child (i.e., the grandchild) was under 16 years of age (*N* = 13,694). Adult children identified by SHARE grandparents as having a child are hereafter referred to as “parents.” Switzerland (*N* = 478) was omitted from our analysis because country-specific indicators were not available. Item missingness was a minor issue: At baseline 841 respondents (6%) were missing one or more of the variables used in the analyses. After deletion of observations with missing data, our final sample consisted of 19,670 parent observations drawn from information on the final sample of 12,375 grandparents, living in 8,546 households, in 10 European countries, with numbers of grandparents ranging from 828 (Denmark) to 1,847 (Belgium).

### Measures

Every grandparent was asked whether they had looked after the grandchildren of each of their adult children in the year prior the interview (“*almost daily*,” “*almost every week*,” “*almost every month*,” or “*less often*”), and how many hours they looked after them (“*on a typical day*,” “*in a typical week*,” “*in a typical month*,” “*in the last 12 months*”). Our outcome of interest was whether parents received “intensive” grandparental childcare. We defined this if grandchildren were looked after by grandparents almost daily or almost every week for at least 15hr a week. This threshold was chosen because, on average, these grandparents looked after their grandchildren 30hr per week roughly equivalent to holding a full-time job (Fuller-Thomson & Minkler, 2001). Preliminary analyses also considered the top quartile of grandparents providing weekly childcare (i.e., at least 12hr per week) but we found no differences in our results. We limited our analyses to grandparental care provided to children under 16 as previous studies have suggested that such help is particularly important for those with school-age children (usually defined as being children in this age group) (Gray, 2005). Across the SHARE countries, 12% of grandparents reported intensive grandchild care to parents whose youngest child was under age 16.

On the basis of the existing literature we identified individual characteristics of grandparents, parents and grandchildren which are known to be associated with the provision of grandparental childcare including age, gender, marital and employment status of both parents and grandparents, education, wealth and health of grandparents, and age of the grandchildren (Fuller-Thomson & Minkler, 2001; Hank & Buber, 2009; Minkler & Fuller-Thomson, 2005). Although separated, never-married, or divorced parents are more likely to use grandparental childcare, it was not possible to distinguish between these categories due to the small numbers: Parents’ marital status was categorised into a binary indicator distinguishing between those who were married/cohabiting and those who were not. Similarly, in our multivariate model because of the small numbers involved in each country, no distinction was made between part-time and full-time workers (less than 60 mothers in Italy and Spain were in part-time work for example). We thus measured the employment status of parents using a dichotomised variable indicating whether or not respondents were in paid work. Likewise, parents with parental leave or homemakers were grouped together as not in paid work (only eight parents were described as homemakers in Denmark). Also for grandparents, data constraints meant it was not possible to include part-time workers; nor to distinguish between being unemployed, a homemaker, or in other work statuses (e.g., self-employed).

To test the extent to which grandparental childcare in Europe was associated with contextual-structural and cultural factors once national distributions in demographic and socio-economic characteristics were taken into account, we included four country-level variables in the model. In particular, the percentage of individuals in a country who believed that a preschool child suffers with a working mother was used as an indicator of societal attitudes toward childcare and women working. This indicator was obtained from the 2008 European Values Study (2011), a cross-cultural survey which collects data on values, attitudes, and norms on a random sample of the adult population across Europe. Because this question was only asked in 1999 and 2008 we chose 2008 data as closer in time to the SHARE data. In order to ensure consistency across the macrolevel indicators used we choose 2008 as the reference year for the other three country-level variables, although there were no substantial differences in these indicators between 2005 and 2008. Possible implications for such a choice are mentioned in the discussion. The percentage of mothers aged 25–49 who were not in paid employment and the percentage of women aged 50–64 in paid-work, were considered to capture the intergenerational labor market structure after preliminary investigation of a number of employment variables. Both indicators were obtained from the 2008 European Union Labour Force Survey (EU-LFS) Eurostat database which collects comparable information for Europe. Finally, the percentage of children under the age of three who were enrolled in formal childcare was used as a country-level indicator of formal childcare provision. We considered enrolment data in both public and private formal childcare to be a more reliable indicator of national childcare practice than number of available places used by Jappens and van Bavel (2012) because usage data captures behavior and includes private childcare which is an important component of childcare regimes in a number of countries (Glaser et al., 2013). This indicator was obtained from the European Union Statistics on Income and Living Conditions (Eurostat, 2008). This survey’s definition of formal childcare includes arrangements such as childcare centres and registered childminders whether public or private.

### Statistical Analyses

Receipt of grandparental childcare as reported by grandparents was modelled using a multilevel logistic regression model. The dataset used is hierarchically structured, with parents (first level), nested into grandparents (second level) who in turn were nested into households (third level), all located across 10 different countries (fourth level). Thus, in our dataset it is possible to study multiple parents receiving grandparental childcare as reported by grandparents. However, the hierarchical data structure violates basic regression assumptions due to the non-independence between observations which may lead to biased estimates, standard errors, and therefore incorrect significance tests (Guo & Zhao, 2000). For this reason, we used a multilevel model permitting us to control for the hierarchical structure of the data—taking into account the parents’ and grandparents’ characteristics, as well as country-level factors—and to adjust for the nonindependence of observations (Goldstein, Browne, & Rasbash, 2002). For example, grandparents who live in the same household are likely to share similar socio-economic and demographic characteristics. However, as in nearly one-third of our sample there was only one grandparent in a household it is computationally challenging to separate the grandparent-level variance component from the household-level variance component. If every household consisted of only one grandparent it would be impossible to separate the two. In order to overcome this problem, we reduced observation sparseness by only considering household level clustering, controlling for the nonindependence of the individual characteristics of grandparents who live in the same household. Thus, we used a third-level random intercept model (reflecting differences between parents, grandparent households and countries) with a dichotomous dependent variable.

Unlike logistic models with only one random error capturing all the variance in the outcome that is unexplained by the model, multilevel models divide the residual variance into three levels, allowing us to capture variation between (i) different parents with the same grandparents; (ii) different grandparent households within the same country, and (iii) different countries. The variance partition also permits us to investigate second and third-level variance; that is, between grandparent households and countries, respectively. Thus, we can say how much of the total variation in grandparental intensive childcare can be attributed to grandparent households or to country-level factors. Multilevel regression models do not provide a direct estimate of first-level variance (parents in our model); for logistic models, the variance at the first level is fixed as the variance of the standard logistic distribution, that is at π^2^/3, or about 3.29 (Goldstein, Browne, & Rasbash, 2002; Snijders & Bosker, 1999).

First, a so-called “empty model” was estimated: This model only includes a random intercept and allows us to detect how much of the total variation in grandparental childcare can be attributed to the different levels (i.e., the household and country-level). Of particular interest in this study is the percentage of the total variation in intensive grandparental childcare that can be attributed to the country-level. Second, parent and grandparent characteristics were considered in order to investigate their effects on grandparental intensive childcare and whether they reduced country-level variation. Finally, country-level variables—centred on the mean values—were included in the model. This allows us to investigate whether the introduction of macrolevel indicators reduces country-level variation. Preliminary analyses were carried out separately for fathers and mothers but, given similarities in the patterns observed, results for both genders combined are presented here. Also, country-level indicators were initially tested one at a time, given the significant correlations between the measures. Each country-level indicator on its own showed a significant association with intensive grandparental childcare. However, we present findings for all four variables considered together. Although this may seem problematic because of the small number of observations at country-level, the robustness of our analyses was confirmed given that the substantive results and direction of association did not change when all measures were included in the model. Moreover, likelihood-ratio chi-square tests indicated that the model with all four country-level predictors fits significantly better than the models including each indicator separately. Finally, although other interactions could have been hypothesised and tested, we decided to examine how individual employment status interacts with the labor market structure because these two measures capture similar information at different levels. We therefore tested—only among mothers—the cross-level interaction between the country-level percentage of mothers aged 25–49 not in paid work and individual-level indicators of employment. Analyses were restricted to respondents with complete data on all variables examined, given the relatively low level of item missingness previously described.

All analyses were performed using Stata, version 12 (Stata Corp, 2011). Maximum-likelihood estimates were derived using the generalised linear latent and mixed models (GLLAMM) adaptive quadrature procedure (Rabe-Hesketh, Skrondal, & Pickles, 2004; Rabe-Hesketh, Skrondal, & Pickles, 2005). We initially used adaptive quadrature with eight quadrature points; however, in line with Rabe-Hesketh and colleagues’ (2004) recommendation, we subsequently refitted the models using 16 quadrature points to assess consistency of estimates. No discrepant values were obtained. For all models, robust standard errors of the estimates are presented as they are more reliable if the data is not normally distributed at each level (Maas & Hox, 2004). Although concerns have been raised about the use of multilevel models with a relatively small number of clusters, recent literature suggests that the estimation of the variance component is accurate even with as little as 10 clusters when estimation procedures based on adaptive quadrature are implemented; similarly, estimates of the regression coefficients tend to be reliable as long as the number of subjects per cluster is greater than 30 (Austin, 2010; Clarke, 2008).

## Results

### Descriptive Statistics

Table 1 shows the percentage of parents receiving grandparental intensive childcare by country. Clear differences across Europe are observed: Overall, around 12% of parents received intensive grandparental childcare, with figures ranging from less than 4% in Denmark and Sweden, to almost one quarter in Greece.

**Table 1.** Percentage (and Absolute Numbers) of Parents With a Child(ren) Who Are Looked After Intensively by a Grandparent, as Well as Mean (and Median) Number of Hours, by Country

	%	*N*	Mean (median)
Denmark	3.6	49/1,316	29.6 (20.0)
Sweden	3.6	100/2,748	31.2 (15.5)
The Netherlands	6.9	164/2,379	29.4 (20.0)
Germany	11.5	209/1,817	24.7 (20.0)
France	11.2	245/2,193	31.1 (24.0)
Austria	12.3	156/1,264	28.3 (20.0)
Belgium	16.3	489/2,992	29.4 (20.0)
Spain	15.2	282/1,854	30.4 (25.0)
Italy	20.3	348/1,717	26.6 (25.0)
Greece	24.8	333/1,341	33.7 (30.0)
Tot SHARE	12.1	2,375/19,670	29.3 (22.0)

Source: SHARE, 2004/5. Unweighted data.

Table 2 presents the frequency distributions of the variables used in our analyses, separately for parents and grandparents. The overwhelming majority of parents were married and in paid work; almost half had two children and around 28% had a youngest child aged 0–2. With respect to grandparents’ characteristics, 77% were married and less than one in five were in paid work. Country differences in the distributions of some key characteristics are striking: For instance, in Sweden, 27% of parents had three or more children compared with just 13% in Italy. The percentage of grandparents in paid work also varied from about 10% in Italy and Spain to more than a third in Sweden and Denmark. Southern Mediterranean grandparents were also relatively older than in other countries.

**Table 2.** Characteristics of Parents and Grandparents in Our Analysis: Descriptive Statistics

	Variables	% SHARE	AT	DE	SE	NL	ES	IT	FR	DK	GR	BE
Parent characteristics	Female	51.9	54.1	52.7	52.8	51.3	52.1	51.9	50.8	51.1	49.5	52.0
	Age											
	<35	35.1	35.2	36.0	36.3	36.0	32.2	34.1	38.3	35.3	29.5	34.8
	35–39	23.9	25.2	26.0	23.5	24.2	24.9	24.6	20.0	23.9	25.5	23.2
	40+	41.0	39.5	38.0	40.1	39.7	42.8	41.3	41.7	40.8	45.0	42.1
	Married	84.8	76.6	79.4	86.7	89.2	91.5	94.4	75.5	70.0	94.4	85.7
	Work status											
	In paid work (full-time)	69.8	62.6	57.1	74.5	55.2	71.2	69.9	77.6	78.9	75.0	74.4
	In paid work (part-time)	11.8	17.4	17.9	10.3	28.0	3.2	4.9	6.8	7.5	4.0	12.8
	Homemaker	9.4	8.2	12.0	1.0	12.4	19.2	20.0	7.9	0.6	15.8	3.9
	Other	9.0	11.8	13.0	14.2	4.4	6.4	5.2	7.7	13.1	5.0	8.8
	*N* of siblings with children <16											
	None	34.9	40.4	43.4	30.0	30.0	31.9	37.8	32.5	30.7	47.6	34.2
	1	40.2	40.6	40.6	44.5	43.1	38.0	38.7	38.4	42.9	39.1	36.6
	2 or 3	24.9	19.0	16.0	25.5	26.9	30.1	23.5	29.1	26.4	13.3	29.2
	Total *N* of children											
	1	31.7	34.0	37.6	24.0	29.9	37.2	42.1	29.1	25.6	30.4	31.7
	2	46.9	46.8	45.9	48.8	46.5	48.2	45.1	42.9	49.1	55.2	44.6
	3 or more	21.3	19.2	16.5	27.2	23.6	14.6	12.8	28.0	25.3	14.4	23.7
	Age of youngest child											
	0–2	27.8	17.4	22.6	29.2	33.9	25.5	26.2	31.5	26.4	25.3	30.7
	3–5	21.6	19.8	20.7	19.9	21.5	23.7	24.3	23.1	23.0	20.6	20.6
	6–11	32.4	37.5	35.4	32.4	29.7	32.8	33.3	28.7	35.5	33.4	30.6
	12–15	18.2	25.3	21.2	18.6	14.9	17.9	16.2	16.8	15.1	20.7	18.1
Grandparent characteristic	Female	56.0	57.6	54.0	53.9	54.6	57.8	58.5	56.9	55.2	60.0	54.8
	Age											
	50–59	26.2	27.2	26.8	28.0	26.2	16.8	21.3	30.5	32.7	19.1	30.1
	60–69	41.3	46.7	47.2	42.4	42.8	40.7	44.8	36.0	39.4	37.9	37.5
	70+	32.5	26.1	26.0	29.6	31.1	42.5	33.9	33.5	27.9	43.0	32.4
	Married	76.4	65.6	81.8	81.6	83.0	79.5	82.5	70.0	66.1	69.4	75.4
	Education											
	High	16.8	20.2	24.2	25.9	14.6	4.3	4.0	14.0	27.7	5.2	22.1
	Middle	26.8	45.2	56.6	19.7	23.4	5.5	9.7	30.4	46.4	14.3	25.5
	Low	56.4	34.6	19.2	54.4	62.0	90.2	86.3	55.6	25.9	80.5	52.4
	Work status											
	In paid work	19.9	14.2	22.4	36.0	17.5	11.1	9.5	20.6	33.1	12.0	171.8
	Retired	55.6	68.9	58.0	58.4	43.6	41.8	60.1	60.7	57.8	53.7	54.9
	Other	24.7	16.9	19.6	5.6	38.9	47.1	30.4	18.7	9.1	34.3	27.3
	With depressive symptoms	25.0	17.1	19.7	17.9	21.2	38.2	34.4	35.1	15.8	30.6	21.9
	Self-rated health = poor or fair	31.2	29.0	39.8	11.9	28.0	46.9	44.8	34.6	26.2	38.1	24.9
	With severe limitations	13.4	12.6	16.1	13.7	20.0	5.6	13.4	14.9	12.0	9.1	12.9
Number of observations												
	Parents	19,670	1,264	1,817	2,748	2,379	1,854	1,717	2,193	1,365	1,341	2,992
	Grandparents	12,375	846	1,252	1,635	1,428	1,121	1,109	1,333	828	958	1,847

Source: SHARE, 2004/5. Unweighted data.

*Note.* Parent characteristics included: (1) marital status using a dichotomised indicator of whether they were married/cohabiting or not (i.e., widowed, divorced/separated, never-married); (2) employment status categorised into a binary indicator distinguishing whether the parent was in paid employment or not. Covariates capturing grandparent characteristics included: (1) educational qualifications using the International Standard Classification of Education (http://www.uis.unesco.org/); (2) wealth quintiles based on the sum of net wealth created by the RAND Corporation (www.mmicdata.rand.org/meta/); (3) marital status using a binary indicator of whether the respondent was married/cohabiting or not or not (i.e., widowed, divorced/separated, never-married); (4) being in paid work, retired or “other” (i.e., “unemployed,” “permanently sick or disabled,” “homemaker,” or “other”).

Table 3 presents the contextual-structural and cultural indicators by country. Considerable variation is observed in the two labor market indicators considered. For instance, in Italy and Greece, where the percentage of intensive grandchild care was highest, just over a third of women aged 50–64 were in paid work compared with close to three quarters in Sweden. Moreover, in those countries more than 40% of mothers aged 25–49 were not in paid work, compared with less than 20% in Denmark and Sweden. The percentage of children under the age of three in formal care also varied considerably, ranging from less than 30% in Italy, Greece and Germany to a high of 73% in Denmark (where the receipt of intensive grandparental childcare was the lowest). Finally, the percentage of people agreeing with the statement that preschool children suffer with a working mother also varied considerably from country to country ranging from 8% in Denmark to 75% in Italy.

**Table 3.** Overview of Cultural-Contextual Factors by Country

Country	Mothers aged 25–49 out of employment %	Women aged 50–64 in paid work %	Children aged 0–2 in formal childcare %	Agreeing that preschool children suffer with working mother %
Denmark	15.2	62.1	73.0	8.0
Sweden	17.0	72.0	49.0	19.5
The Netherlands	21.0	53.4	47.0	39.0
Germany	29.0	56.4	19.0	50.0
France	25.0	49.8	40.0	42.0
Austria	24.5	46.8	29.0	64.7
Belgium	24.7	38.9	35.0	38.4
Spain	37.0	39.6	39.0	48.0
Italy	44.0	34.8	27.0	75.0
Greece	40.4	35.9	16.0	72.5

Source: Eurostat Statistics on Income and Living Conditions, 2008; Eurostat Labour Force Survey, 2008; European Values Study, 2008.

### Multilevel Model

Combining all of our explanatory indicators, Table 4 shows the results of five multilevel models. Model 1 includes only the basic demographic parent characteristics of gender and age; model 2 adds the other selected parent characteristics; model 3 adds grandparent’s characteristics; model 4 adds country-level variables; and model 5 considers cross-level interactions between mothers’ employment and the general level of employment in the country. Although we refer to “a grandparent” when presenting results, it is important to bear in mind that—given that information on childcare is obtained from the grandparents—we only know whether the parents received childcare from either their mother or father, but not their parents-in-law. In this section, we focus on describing results for model 4, as caution is needed when comparing odds ratios across nested models as the first-level variance is fixed in logistic multilevel regression as noted previously (Mood, 2010).

**Table 4.** Multilevel Models Predicting Parents With a Child Looked After Intensively by a Grandparent (10 Countries)

	Model 1	Model 2	Model 3	Model 4	Model 5
	Odds ratios (SE)	Odds ratios (SE)	Odds ratios (SE)	Odds ratios (SE)	Odds ratios (SE)
Parent’s characteristics					
Female	2.377 (0.188)***	3.075 (0.268)***	3.142 (0.281)***	3.139 (0.280)***	
Age (reference < 35): 35–39	0.809 (0.076)**	1.007 (0.103)	0.951 (0.104)	0.954 (0.104)	0.710 (0.119)**
40+	0.242 (0.025)***	0.469 (0.057)***	0.494 (0.067)***	0.496 (0.068)***	0.367 (0.056)***
Not married (reference: married/cohabiting)		2.211 (0.250)***	2.375 (0.276)***	2.376 (0.274)***	3.789 (0.674)***
In paid work (reference: not in paid work)		2.078 (0.228)***	2.054 (0.232)***	2.060 (0.232)***	2.650 (0.315)***
Without siblings with children < 16		1.688 (0.161)***	1.822 (0.181)***	1.821 (0.180)***	2.525 (0.367)***
Number of children (reference: 1): 2		1.095 (0.097)	1.072 (0.097)	1.083 (0.098)	1.008 (0.141)
3 or more		0.746 (0.094)**	0.739 (0.095)**	0.745 (0.096)**	0.574 (0.118)***
Age youngest child (reference: 0–2): 3–5		1.347 (0.143)***	1.372 (0.149)***	1.375 (0.149)***	1.232 (0.121)*
6–11		0.830 (0.093)*	0.825 (0.094)*	0.830 (0.095)	0.639 (0.107)**
12–15		0.243 (0.039)***	0.241 (0.040)***	0.242 (0.040)***	0.151 (0.039)***
Grandparent’s characteristics					
Female			2.025 (0.171)***	2.023 (0.171)***	2.629 (0.309)***
Age (reference: 50–59): 60–69			1.053 (0.129)	1.057 (0.129)	1.062 (0.180)
70+			0.638 (0.104)***	0.645 (0.104)***	0.644 (0.153)***
Married (reference: unmarried)			1.747 (0.214)***	1.741 (0.213)***	2.495 (0.461)***
Level of Education (reference: low): Middle			0.749 (0.083)***	0.755 (0.082)***	0.954 (0.175)
High			0.793 (0.107)*	0.813 (0.108)	1.173 (0.225)
Employment status (ref: retired): in paid work			0.542 (0.074)***	0.556 (0.075)***	0.486 (0.093)***
Other			0.818 (0.090)*	0.822 (0.088)*	0.788 (0.125)*
In lowest wealth quintile			0.862 (0.113)	0.863 (0.114)	0.937 (0.184)
Health characteristics (ref: no such problems): Depressed			0.968 (0.099)	0.962 (0.099)	0.919 (0.139)
SHR= poor or fair			0.923 (0.092)	0.921 (0.092)	0.832 (0.119)*
In lowest cognitive quintile			0.685 (0.091)***	0.687 (0.091)***	0.585 (0.112)***
Severe functional limitations			0.785 (0.110)**	0.776 (0.101)**	0.834 (0.171)
Country-level Characteristics					
Mothers 25–49 not in paid work				1.017 (0.005)**	1.010 (0.024)
Women 50–64 in paid work				0.940 (0.007)***	0.929 (0.013)***
Formal Childcare (0–2)				0.974 (0.008)***	0.979 (0.011)**
Child suffers with working mother				1.014 (0.013)	0.999 (0.014)
“Mother in paid work” * “Mothers 25–49 not in paid work”					1.063 (0.017)***
Constant	0.026 (0.010)***	0.008 (0.004)***	0.005 (0.002)***	0.005 (0.001)***	0.006 (0.002)***
Grandparent household level variance	6.143 (0.456)	6.094 (0.489)	5.743 (0.455)	5.748 (0.454)	5.982 (0.503)
Country-level variance	1.539 (0.642)	1.489 (0.686)	1.428 (0.661)	0.157 (0.066)	0.203 (0.043)
Country-level variance as % of total variance	14.0%	13.7%	13.6%	1.7%	2.1%
Log likelihood	−6,150.77	−5,497.7	−5,402.87	−5,281.00	−3,322.91
Number of observations (*N*)	19,670	19,670	19,670	19,670	10,205

Sources: SHARE 2004/5; Eurostat Statistics on Income and Living Conditions, 2008; Eurostat Labour Force Survey, 2008; European Values Study, 2008. Own calculations.

*Notes.* SE = standard error.

*p < .10, **p < .05, ***p < .01.

Covariates capturing grandparent characteristics included: (1) educational qualifications using the International Standard Classification of Education where a low educational level is defined as being below a secondary education, and high refers to university education or above (http://www.uis.unesco.org/); (2) wealth quintiles based on the sum of the net value of properties, nonhousing financial wealth, and business assets created by the RAND Corporation (www.mmicdata.rand.org/meta/); (3) marital status using a binary indicator of whether the respondent was married (either in a legal or cohabiting union) or not (i.e., widowed, divorced/separated, never-married); (4) being in paid work, retired or “other” (i.e., “unemployed,” “permanently sick or disabled,” “homemaker,” or “other”); and (5) health, assessed using a variety of indicators, including cognitive index quintiles, self-rated health, depressive symptomatology and functional limitation. Cognitive ability was assessed by combining several questions relating to “orientation in time,” “word recall,” “verbal fluency,” and “numeracy” skills, as described in Mazzonna and Peracchi (2012). Self-rated health (SRH) was measured on a five-point ordinal scale (*excellent*, *very good*, *good*, *fair*, or *poor*). The five SRH items were dichotomised into “fair or poor” versus better health. Respondents who reported four or more symptoms on the EURO-D 12-item scale were classified as reporting depressive symptomatology (Prince et al., 1999). Functional health was measured as having any long-term health problems which severely limiting the respondent’s activities. Covariates capturing parent characteristics included: (1) marital status using a dichotomised indicator of whether they were married/cohabiting or not; (2) employment status categorised into a binary indicator distinguishing whether the parent was in paid employment or not; and (3) presences of siblings whose youngest child was younger than 16.

Results show that mothers under the age of 40 and who were unmarried were more likely to have a child looked after by a grandparent intensively. Similarly, parents who were in paid work (either full- or part-time) were more likely to receive grandparental childcare compared with those not in paid work. Our results also suggest that parents who did not have a sibling with children were significantly more likely to have a child looked after intensively by a grandparent. If a parent had three or more children, this reduced the odds of any of their children being looked after intensively by a grandparent; and parents whose youngest child was aged between three and five were significantly more likely to have their child looked after intensively by grandparents than those whose youngest child was under three. Parents whose youngest child was aged between 12 and 15 were significantly less likely to have a child receiving such care from their grandparents.

As for grandparents’ characteristics, grandmothers were more likely to care intensively for grandchildren than grandfathers, and if grandparents were younger, married, and with low levels of education. Grandparents in paid work were significantly less likely to look after grandchildren intensively compared with those not in paid work. Finally, grandparents in the lowest cognitive quintile, or who reported a limiting long-term illness, were significantly less likely to look after grandchildren intensively.

Model 4 also includes aggregated country characteristics. This shows that once individual factors are controlled, at country-level, as the percentage of women aged 50–64 in paid work increases, the likelihood of grandparental intensive childcare decreases; whereas as the percentage of mothers aged 25–49 not in paid work in a country increases, the odds of receiving intensive grandparental childcare also increases. Formal childcare and grandparental childcare seem to some extent to be substitutes: A parent is more likely to get intensive grandparental help as the percentage of children aged 0–2 not in formal care increases. Finally, there was virtually no association between the societal level of disapproval of mothers with preschool children working and intensive grandparental childcare when all four country-level variables were considered, suggesting that these cultural factors are already captured and reflected by the employment and childcare environment. Model 5 explores the cross-level interaction between mother’s employment and the country-level indicator of the employment rate among mothers aged 25–49. Results suggest that mothers are indeed more likely to have their children looked after by a grandparent if in paid work, but this becomes even more likely as the percentage of mothers not in paid employment in the country increases.

The model divides the total variance of the outcome variable between the three levels (i.e., parent, grandparental household, and country-levels representing first, second, and third levels, respectively). The statistics reported at the bottom of Table 4 present the variance estimates for the second and third levels only (the first-level variance, here defined as parent level, is fixed at 3.29 as discussed previously). This statistic is the same as the residual intraclass correlation (Snijders & Bosker, 1999). We are particularly interested in observing whether and how differences across countries in intensive grandparental childcare decrease when individual and family characteristics, as well as macroindicators are included in the model. In Model 1, although differences between households were larger than differences between countries (as indicated by the variance estimates at the bottom of Table 4), country membership still accounted for 14% of the total unexplained variance. Models 2 and 3, which included parent and grandparent characteristics, respectively, show a reduction in household or second-level variance (more substantial when grandparents’ characteristics are accounted for); although no reduction in country-level variance was observed (i.e., around 14% of the total variation still remains unexplained). The introduction of the country-level contextual-structural, and cultural factors, however, considerably reduced country-level variance in Model 4 to less than 2% of the total residual variance. This reveals therefore that it is a country’s labor market structure and formal childcare provision, rather than compositional demographic and socio-economic differences, which capture most of the cross-country variation in intensive grandparental childcare.

## Discussion

Our analyses indicate that the provision of intensive childcare support to parents by grandparents varies considerably across European countries. Our multilevel study aimed to investigate the extent to which such variation in intensive grandparental childcare may be explained by national variations in demographic and socio-economic characteristics of parents, grandchildren, and grandparents and/or by contextual-structural and cultural factors. Demographic and socio-economic characteristics of both parents and grandparents vary dramatically across European countries, suggesting that some of the observed variations in the prevalence of intensive grandparental childcare may well be accounted for by such differences. For instance, parents are more likely to be married, older, and to have just one child in Italy, Greece and Spain, where a higher percentage have their children looked after intensively by grandparents. Similarly, the composition of grandparents varies across the countries under study, with Italian, Greek, and Spanish grandparents more likely to have a lower level of education, and not be in paid work.

However, this study has shown that variations across countries in the prevalence of these characteristics explain relatively little of the cross-national variation in intensive grandparental childcare. Our analysis shows that the country-level variation in intensive grandparental childcare observed in the European countries studied is mostly explained by differences in a country’s female labor market structure across age groups and formal childcare provision.

Although recent comparative studies suggested that welfare policies do play a role in shaping grandparental childcare provision (Albertini, Kohli, & Vogel, 2007; Igel & Szydlik, 2011; Jappens & van Bavel, 2012), few studies have accounted for country-level variables; furthermore, focus has hitherto largely been limited to public investments in child-care infrastructures (Igel & Szydlik, 2011) and cultural attitudes to gender roles (Jappens & van Bavel, 2012) rather than wider childcare usage, and attitudes to childcare in particular. Although policy theorists have focused heavily on how policy environments affect maternal childcare (Daly, 2000; Keck & Saraceno, 2013; Nieuwenhuis, Need, & Van Der Kolk, 2012; Orloff, 2002), we argue that an intergenerational approach is critical when country-level indicators are considered; in particular, that the labor force participation of both mother and grandmother generations in the workforce needs to be taken into account.

Our multivariate multilevel analyses reinforce the hypothesis that contextual-structural factors from the perspective of both generations are critical for understanding variations in grandparental childcare. Our findings suggest that the odds of parents receiving intensive childcare support from grandparents decreases as the percentage of mothers and older women in paid employment increases. Extensive formal (public and private) childcare seems to offset intensive grandparental childcare, in line with previous studies (Attias-Donfut, Ogg, & Wolff, 2005; Hank & Buber, 2009; Igel & Szydlik, 2011; Koslowski, 2009): As the percentage of formal childcare provision in a country increases, parents are less likely to receive intensive grandparental childcare.

In countries where both mothers and grandmothers are not expected to be in paid work (i.e., where part-time opportunities and parental leave benefits for working mothers are restricted), and where formal childcare opportunities are limited, we find higher odds of intensive grandparental childcare, even though there are higher proportions of mothers at home. Indeed, our findings suggest that the likelihood of receiving intensive grandparental childcare is not only associated with country and individual-level factors, but also with their interaction. The odds of a mother receiving intensive childcare by grandparents are associated with the individual working status of the mother as well as with the country level of employment among mothers. If mothers do engage in paid work in countries where they are not expected to be employed but to look after children, reliance on grandparental support is considerable. It would seem that where maternal paid work is not the norm, there are fewer childcare choices available to women in paid work, and/or in those countries preferences for within-family childcare are strong.

At an individual level, our results are in line with previous studies and show that younger mothers in paid work and those who are not married were more likely to have a child looked after intensively by a grandparent, particularly by grandmothers who are younger, married, in good health and not in paid work (Fuller-Thomson & Minkler, 2001; Hank & Buber, 2009; Herlofson & Hagestad, 2012; Koslowski, 2009; Vandell et al., 2003; Wheelock & Jones, 2002; Zamarro, 2011). Also, parents in our study were more likely to receive grandparental assistance if they had no siblings with young children. This may be because having siblings with young children makes grandparents’ availability scarcer, as grandparents may already provide intensive childcare to siblings (Aassve, Meroni, & Pronzato, 2012). However, unlike Igel and Szydlik’s (2011) study which found that regular grandparental childcare was more likely for children under 3 years of age and less likely for children aged 6–12, in our study we found that once other factors were controlled for, parents were more likely to have a child looked after intensively by a grandparent if their youngest child was preschool age, in particular between the ages of three and five. We found no statistically significant differences between parents whose youngest children were infants, aged 0–2, and those whose youngest was aged 6–11. Given that the percentage of children aged 3–5 enrolled in preschool services is above 80% in Europe, with practically universal coverage in Belgium, France, and Spain (OECD, 2012), and enrolment in primary school is above 95% further work may need to account for the number of hours children attend formal preschool childcare and primary school.

### Strengths, Limitations and Implications

Contributions of the study include an intergenerational approach using multilevel analyses, which explicitly examine the association between intensive grandparental childcare and cross-national differences in the demographic and socio-economic characteristics of children, parents, and grandparents and in labor market structures, formal childcare provision, and cultural expectations regarding paid work among mothers with young children. Our findings suggest that despite cross-national variation in distributions of demographic and socio-economic characteristics, labor force participation of women of different ages, as well as formal childcare usage, are key explanatory factors for national variations in intensive grandparental childcare.

Nevertheless, our analysis has some limitations. First, the measurements considered are based on self-reports; for example, the intensity and frequency of grandchild care or self-rated health. This may be problematic as it could be sensitive to cultural differences in definitions (Jylhä, Guralnik, Ferrucci, Jokela, & Heikkinen, 1998). Second, information on intensive grandparental childcare and individual characteristics of parents are based on grandparents’ reports. Third, the SHARE questionnaire provided no detailed information on the nature of the work undertaken by parents. This is important because we know that parents who work nights, weekends, or nonstandard hours require a higher intensity of grandparental childcare (Vandell et al. 2003). Fourth, our study did not examine the effect of multiple-role commitments by grandparents, as looking after grandchildren intensively may compete with other forms of support, such as caring for spouses or parents. Similarly, it is not known whether parents also use other forms of either formal or informal childcare, and to which extent they do so. Fifth, as the data are cross-sectional, the experiences described may be unique to this particular period and to the cohorts considered. However, as female labor force participation is likely to increase we may find a stronger relationship between employment status and receipt of intensive grandparental childcare in the future. Moreover, as the microlevel and macrolevel data used predated the recession which started in 2008, we are unable to assess the impact of the economic downturn on grandparental childcare. Finally, although this study contributes to our knowledge of associations between structures, institutions, values, and family solidarity in the form of grandparent childcare, disentangling the links between individual behaviors, welfare systems, and norms is complex, as these are all multifaceted relationships which are rooted and embedded in society and culture (van Oorschot, Opielka, & Pfau-Effinger, 2008).

Our study, nonetheless, suggests that parents—and particularly working mothers—tend to rely more on grandparental childcare in those countries with limited provision of childcare and where mothers and grandmothers are not encouraged to participate in the labor market. This has important policy implications because among the main aims of the Lisbon Strategy, which remains central to the EU’s 2020 Agenda (http://ec.europa.eu/europe2020), is the promotion of employment growth (particularly among women) and the extension of working lives. This is likely to affect the availability of grandparents to provide grandchild care, which in turn might create a care gap for working parents, potentially impacting on mothers’ employment. Indeed, grandparents whom governments across Europe are seeking to retain in the labor market (European Commission, 2010) are the very men and women in their 50s and 60s who are the most likely to be providing intensive childcare, that is to be looking after their grandchildren almost daily and about 30hr per week on average. Such incompatibility between full-time employment and provision of intensive grandchild care might potentially affect the labor participation of young mothers particularly in Southern European countries where there is currently little formal childcare, unless other concurrent policies were implemented.

## Funding

This paper uses data from SHARE Wave 1 Release 2.5.0, as of May 24, 2011. The SHARE data collection has been primarily funded by the European Commission through the 5th Framework Programme (project QLK6-CT-2001-00360 in the thematic programme Quality of Life), through the 6th Framework Programme (projects SHARE-I3, RII-CT-2006–062193, COMPARE, CIT5-CT-2005–028857, and SHARELIFE, CIT4-CT-2006–028812), and through the 7th Framework Programme (SHARE-PREP, N° 211909, SHARE-LEAP, N° 227822 and SHARE M4, N° 261982). Additional funding from the U.S. National Institute on Aging (U01 AG09740-13S2, P01 AG005842, P01 AG08291, P30 AG12815, R21 AG025169, Y1-AG-4553-01, IAG BSR06-11, and OGHA 04-064) and the German Ministry of Education and Research as well as from various national sources is gratefully acknowledged (see www.share-project.org for a full list of funding institutions). This research was supported by the Calouste Gulbenkian Foundation in partnership with Grandparents Plus and the Beth Johnson Foundation which funded the research undertaken at King’s College London.
